# Dual stoichiometry and subunit organization in the ClpP1/P2 protease from the cyanobacterium *Synechococcus elongatus*

**DOI:** 10.1016/j.jsb.2015.10.016

**Published:** 2015-12

**Authors:** Victor A. Mikhailov, Frida Ståhlberg, Adrian K. Clarke, Carol V. Robinson

**Affiliations:** a Department of Chemistry, Physical and Theoretical Chemistry Laboratory, University of Oxford, Oxford, UK; b Department of Biological & Environmental Sciences, Gothenburg University, Gothenburg, Sweden

**Keywords:** Clp protease, Enzyme structure, Macromolecular protein complexes, Structural biology, Mass spectrometry (MS), Stoichiometry

## Abstract

The Clp protease is conserved among eubacteria and most eukaryotes, and uses ATP to drive protein substrate unfolding and translocation into a chamber of sequestered proteolytic active sites. To investigate the proteolytic core of the ClpXP1/P2 protease from the cyanobacterium *Synechococcus elongatus* we have used a non-denaturing mass spectrometry approach. We show that the proteolytic core is a double ring tetradecamer consisting of an equal number of ClpP1 and ClpP2 subunits with masses of 21.70 and 23.44 kDa, respectively. Two stoichiometries are revealed for the heptameric rings: 4ClpP1 + 3ClpP2 and 3ClpP1 + 4ClpP2. When combined in the double ring the stoichiometries are (4ClpP1 + 3ClpP2) + (3ClpP1 + 4ClpP2) and 2 × (3ClpP1 + 4ClpP2) with a low population of a 2 × (4ClpP1 + 3ClpP2) tetradecamer. The assignment of the stoichiometries is confirmed by collision-induced dissociation of selected charge states of the intact heptamer and tetradecamer. Presence of the heterodimers, heterotetramers and heterohexamers, and absence of the mono-oligomers, in the mass spectra of the partially denatured protease indicates that the ring complex consists of a chain of ClpP1/ClpP2 heterodimers with the ring completed by an additional ClpP1 or ClpP2 subunit.

## 1 Introduction

Proteases play a key role in maintaining the protein environment, or proteostasis of all cells. Much of the selective protein degradation is performed by AAA+ (ATPases associated with various cellular activities) proteases, the best studied of which include the 26S proteasome in eukaryotes and the Clp proteases in eubacteria. These two principle proteases have a similar two-component architecture and mode of action. The typical bacterial Clp protease, such as that from *Escherichia coli* consists of a proteolytic ClpP core flanked on one or both sides by the ATP-dependent chaperones ClpA or ClpX (Grimaud et al., 1998). The barrel-shaped ClpP core is comprised of two opposing heptameric rings. Each ClpP subunit within this structure has the active site positioned inside and contains the catalytic triad of Ser, His and Asp residues common to Ser-type proteases. Due to narrow entries at either end, the ClpP core alone can only degrade short unstructured peptides and relies on its chaperone partners to degrade larger native protein substrates (Thompson and Maurizi, 1994). Once the Clp ATPases bind the targeted substrate, they unfold the protein and translocate it into the ClpP proteolytic chamber (Kim et al., 2001, Ortega et al., 2002). The unfolded substrate once inside the core complex is bound in an extended manner to different ClpP subunits and degraded into small peptides that appear to diffuse out through lateral gaps (Kang et al., 2004).

The Clp protease exists in many different organisms from all eubacteria to mammalian and plant mitochondria, and in plastids of algae and plants. In most eubacteria, the proteolytic core consists of a single type of ClpP protein, but in certain Gram-positive species such as *Streptomyces lividans* and *Mycobacterium tuberculosis* additional paralogs exist (Viala et al., 2000, Engels et al., 2004). Multiple ClpP paralogs is also a common feature in cyanobacteria, with most possessing three (ClpP1–3) as in the model species *Synechococcus elongatus* (hereafter referred to as *Synechococcus*). Cyanobacteria also contain a variant of ClpP, now known as ClpR that has protein sequence similarity to ClpP but lacks the catalytic triad of Ser-type proteases and is proteolytically inactive (Andersson et al., 2009). This complexity of Clp proteins in cyanobacteria diversifies further in plastids of algae and plants, with the Clp proteolytic core in *Arabidopsis thaliana* consisting of five ClpP and four ClpR paralogs (Peltier et al., 2004).

We have shown that two distinct Clp proteases exist in *Synechococcus*, both of which contain mixed proteolytic cores. The first consists of ClpP3 and ClpR subunits, and associates with ClpC, whereas the other has a proteolytic core consisting of ClpP1 and ClpP2 that binds to ClpX (Stanne et al., 2007). A recombinant version of the *Synechococcus* ClpP3/R was purified by co-overexpression in *E. coli* and shown to be proteolytically active in association with *Synechococcus* ClpC. Structural analysis of the ClpP3/R core complex by mass spectrometry revealed two identical heptameric rings, each containing three ClpP3 and four ClpR monomers arranged in an alternating configuration (i.e., R-P3-R-P3-R-P3-R) (Andersson et al., 2009).

In this study, we have now purified the *Synechococcus* ClpP1/P2 core complex using a similar expression system in *E. coli* to that employed for ClpP3/R. Using various mass spectrometry techniques in combination with solution denaturation (Hernandez et al., 2006, Hilton and Benesch, 2012) we reveal that the ClpP1/P2 heptameric rings have a configuration where a chain of three ClpP1/ClpP2 dimers forms a seven fold ring completed by either a ClpP1 or ClpP2 subunit. Furthermore, and distinct from the ClpP3/R complex, more than one type of ring conformation can be observed leading to a wider diversity of structures.

## 2 Materials and methods

### 2.1 Over-expression of *Synechococcus* ClpP1 and ClpP2

The *Synechococcus clpP1* and *clpP2* genes were amplified from genomic clones using Pfx DNA polymerase (Life Technologies) and cloned into the pCDF and pCOLA Duet vectors, respectively (Novagen) for co-expression in *E. coli*. A His_6_ tag was added to the 3′ end of the *clpP2* gene to aid purification, whereas no such tag was included in the *clpP1* gene. The integrity of the final construct was confirmed by sequencing. Co-expression of ClpP1 and ClpP2 was performed in *E. coli* BL21 cells in which the native *clpP* gene was inactivated. Cultures were grown in LB at 37 °C, and once reaching mid-exponential growth (i.e. A_600_ of 0.5) isopropyl 1-thio-β-d-galactopyranoside was added (final concentration 0.4 mm) to induce protein expression. Cells were pelleted after 2 h and then resuspended in buffer A (20 mM Tris/Cl pH 7.5, 300 mM NaCl, 40 mM imidazole, 1 mM DTT). Cells were ruptured using a French Press (1000 atm) followed by centrifugation to remove insoluble cell debris. The soluble protein fraction was loaded onto a Ni^2+^ affinity column (HisTrap HP, GE Healthcare). After washing the column with buffer A, bound proteins were eluted using buffer B (20 mM Tris/Cl pH 7.5, 300 mM NaCl, 400 mM imidazole, 1 mM DTT). Denaturing-PAGE revealed that both ClpP1 and ClpP2 were purified in the same fraction, indicating that the co-expressed proteins formed a single stable oligomer. The ClpP1/P2 oligomer was further purified by gel filtration chromatography using a 16/60 Superdex column (GE Healthcare). Proteins were stored in buffer C (20 mM Tris/Cl pH 7.5, 75 mM NaCl, 1 mM DTT) with 20% glycerol. Protein concentration was determined using the Bradford assay (Thermo Scientific), with the concentration of ClpP1/P2 used in each assay based on a monomeric conformation.

The recombinant ClpP1/P2 proteins were prepared in NuPAGE sample buffer (final concentration: 50 mM Tris–HCl pH 8.5, 2% lithium dodecyl sulfate, 10% glycerol, 0.5 mM EDTA, 50 mM DTT) and denatured at 75 °C for 10 min. Native cell protein extracts were isolated as previously described (Stanne et al., 2007) from cultures of wild type *Synechococcus* grown under standard conditions to early exponential phase (3.0–3.5 μg Chl/ml). Native protein extract (0.1 μg Chl) and recombinant ClpP1/P2 (2–3 ng protein) were separated by denaturing-PAGE on 12% Bis-Tris NuPAGE gels (Life Technologies), with ClpP1 and ClpP2 then detected by immunoblotting with the specific polyclonal antibodies (Clarke et al., 1998, Schelin et al., 2002). The amino acid composition of the processed form of recombinant ClpP2 was determined by mass spectrometry. Analysis was performed on semitrypitc-treated ClpP2 protein that had been separated on denaturing-PAGE and then excised. The sequence coverage was 77.5% based on peptide sequences identified with at least 95% confidence.

The oligomeric state of the recombinant ClpP1/P2 was determined by colorless native PAGE (CN-PAGE), where 5 μg of protein was separated on 4–13% polyacrylamide gels using the Tris–Borate system (Stanne et al., 2007). As previously described, proteins were electrophoresed until they had reached their pore limitation within the gel matrix for more accurate size determination of the native protein complexes (Stanne et al., 2007). Proteins were visualized by colloidal coomassie blue staining.

### 2.2 GFP-SsrA-degradation assay

Activity of the purified ClpP1/P2 complex was tested by performing a degradation assay with the *E. coli* ClpX (EcClpX) chaperone and its model substrate GFP-SsrA. The assay was performed in buffer D (20 mM Tris HCl pH 7.5, 25 mM NaCl, 150 mM KCl, 4 mM MgCl_2_, 1 mM DTT) and in the presence of an ATP-regeneration system (Dougan et al., 2002). EcClpX (0.9 μM) was added in excess of ClpP1/P2 (0.3 μM). The assay was performed at 37 °C and samples were collected at different time points, which were treated with NuPAGE sample buffer and denatured at 75 °C for 10 min. Samples were separated on 12% Bis-Tris gels (Life Technologies) and visualized by staining with colloidal coomassie blue. The amount of GFP-SsrA at each time point was quantified using the ChemiGenius2 imaging system (Syngene) and associated software.

### 2.3 Mass spectrometry

Ions of ClpP1/P2 protease for mass analysis were generated using a nano-electrospray ionization (nESI) introduction system on a quadrupole time-of-flight (qToF) mass spectrometer (Q-ToF 2, Waters/Micromass, Manchester, UK) modified for high masses (Nettleton et al., 1998, Sobott et al., 2002). To provide non-denaturing solution for the electrospray, the initial solution of ClpP1/2 after purification was buffer exchanged into 200 mM ammonium acetate solution using BioSpin 6 microcolumns (Bio-Rad Laboratories). The following MS parameters were used: capillary voltage 1.55–1.71 kV, sample cone voltage 100–200 V, extractor cone voltage 50–100 V, ion guide pressure in the range of 2.5–6.5 × 10^−3^ mbar. Collision induced dissociation (CID) was carried out in the collision cell of the instrument, with 50–200 V voltage and 10^−3^ mbar argon. All spectra were calibrated externally using CsI solution (50 mg/ml). Spectra are presented here with minimal smoothing.

Mass spectra of ClpP1/P2 protease were obtained using non-denaturing or partly denaturing solution conditions. The stoichiometries of the ClpP1/P2 complexes were determined from the values of molecular mass obtained from the *m/z* of the charge state series observed in mass spectra. Experimental molecular masses were then compared with theoretical values for possible assignments. A tolerance of 0.1–0.4 kDa was set, this value being less than the difference in mass between ClpP1 and ClpP2 (1.74 kDa). Further confirmation of the stoichiometry was obtained using collision induced dissociation (CID) technique. Ions of the oligomer of interest in a particular charge state (or several states) were isolated in the quadrupole of the mass spectrometer and subjected to collisions with Ar atoms. This procedure induced loss of a single protein subunit, ClpP1 or ClpP2, from the precursor complex. The masses of the ejected protein subunits and the remaining stripped oligomeric complex were then deduced to confirm the assignment of the original complex as described elsewhere (Benesch et al., 2007).

Partial denaturation of the protease complex was carried out by adding aliquots of denaturing agents (triethylamine acetate (TEA) and ammonium hydroxide) to the protein solution prior to MS analysis. Under certain conditions the complex dissociated to form sub-oligomers assigned to specific combinations of ClpP1 and ClpP2 subunits. This approach reveals how the two types of subunits are arranged within the ring complex.

## 3 Results

### 3.1 Purification of ClpP1/P2 complex

**Fig. 1:**
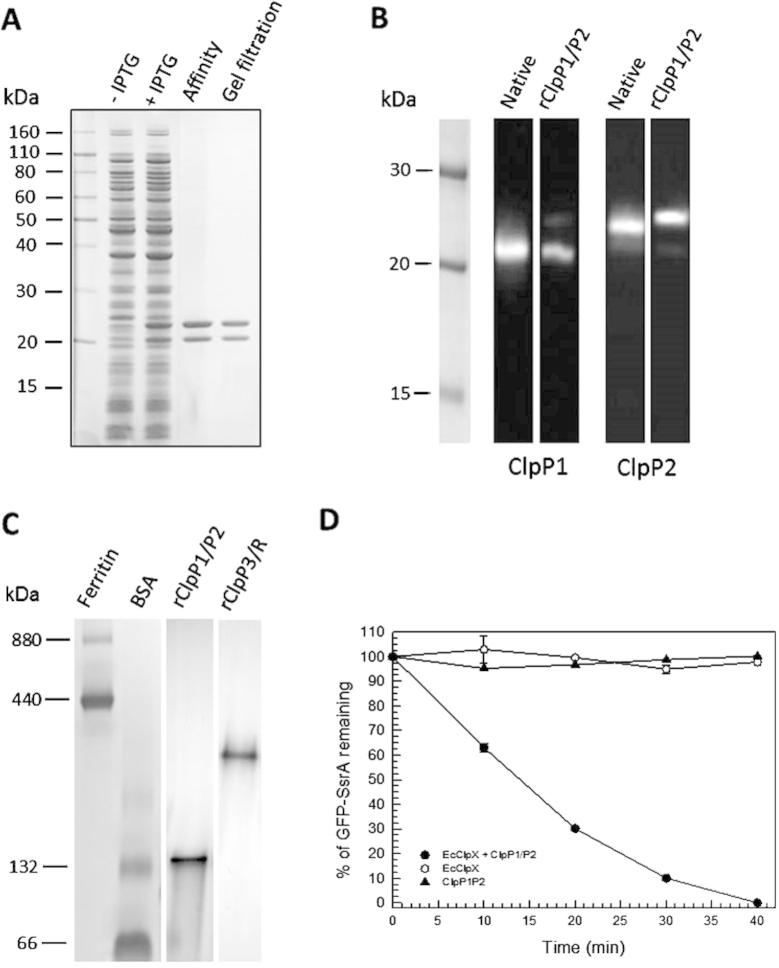
Purification of recombinant ClpP1/P2 core complex. (A) Native ClpP1 and C-terminal His-tagged ClpP2 were co-expressed in *E. coli* upon induction with IPTG. ClpP1/P2 was purified from soluble cell extracts sequentially by Ni^2+^-affinity chromatography and gel filtration, with ClpP1 (21 kDa) and ClpP2 (23 kDa) visualized by SDS–PAGE and Coomassie Blue staining. (B) Size comparison between native and recombinant ClpP1 and ClpP2 proteins. Purified rClpP1/P2 (2–3 ng protein) along with a cell protein extract (0.1 μg Chl) from wild type *Synechococcus* were separated by SDS–PAGE and detected immunologically using specific antibodies for ClpP1 and ClpP2 (Clarke et al., 1998, Schelin et al., 2002, Stanne et al., 2007). (C) Oligomeric size of rClpP1/P2. Purified ClpP1/P2 (5 μg) along with purified *Synechococcus* ClpP3/R were separated by native-PAGE and visualized by Coomassie Blue staining. Size of the ClpP1/P2 heptameric rings (about 140 kDa) was determined from molecular mass standards (ferritin [440 kDa monomer, 880 kDa dimer] and BSA [66 kDa monomer, 132 kDa dimer]) as shown on the left. (D) Activity of the rClpP1/P2 proteolytic core. Degradation of GFP-SsrA (2 μM) by rClpP1/P2 with *E. coli* ClpX. The remaining amount of GFP-SsrA was quantified relative to the time 0 control, which was set to 100%. Shown are mean values ± S.E.M. (*n* = 3).

We have previously shown that the two constitutively expressed ClpP1 and ClpP2 proteins in *Synechococcus* appear to associate in one oligomeric complex *in vivo* (Stanne et al., 2007). To purify this ClpP1/P2 complex, we used a similar *E. coli* expression system that successfully purified the *Synechococcus* ClpP3/R core complex (Andersson et al., 2009). Based on the premise that both proteins would readily oligomerize together once synthesized in *E. coli*, a His_6_ tag was included at the C terminus of the ClpP2 subunit only to aid in purification of the core complex. Upon induction with isopropyl 1-thio-d-galactopyranoside, recombinant ClpP1 and ClpP2 (rClpP1 and rClpP2, respectively) in soluble cell extracts were isolated by Ni^2+^-affinity chromatography. ClpP1 and ClpP2 were the principle proteins eluted from the column indicating they form a stable oligomeric complex. The ClpP1/P2 proteins were then further purified by gel filtration (Fig. 1A).

To determine whether the rClpP1 and rClpP2 proteins corresponded to those *in vivo* we first separated the purified proteins by denaturing-PAGE and performed immunoblotting. Using antibodies specific for either protein (Clarke et al., 1998, Schelin et al., 2002) rClpP1 was shown to have the identical monomer size to the native ClpP1 protein (*i.e.*, 21 kDa). Taking into account the addition of the His_6_ tag, rClpP2 also matched the size of native ClpP2 (Fig. 1B). Analysis by mass spectrometry revealed that rClpP2 had autolytically processed its N-terminus to produce the correct mature protein (experimental mass of 23.4 kDa, as predicted for the processed protein, and opposed to theoretical mass of 26.8 kDa for the unprocessed protein with His_6_ tag (Schelin et al., 2002)). When resolved by native-PAGE, the purified rClpP1/P2 proteins formed a single oligomeric complex of ca. 140 kDa (Fig. 1C) corresponding to the single heptameric ring of native ClpP1/P2 as previously shown (Stanne et al., 2007). In our previous study native ClpP1/P2 oligomers corresponding to the size of a double-ring tetradecamer were observed by size exclusion chromatography, suggesting that it is the electrophoresis conditions that compromise the tetradecamer stability for both wild type and recombinant proteins (Stanne et al., 2007). In this work the presence of rClpP1/ClpP2 tetradecamers has been confirmed by non-denaturing mass spectrometry (see below), indicating that the His_6_ tag does not disturb their formation. The rClpP1/P2 proteins form a proteolytically active complex in association with their chaperone partner ClpX, as shown by degradation of the model substrate GFP-SsrA (Fig. 1D).

**Fig. 2:**
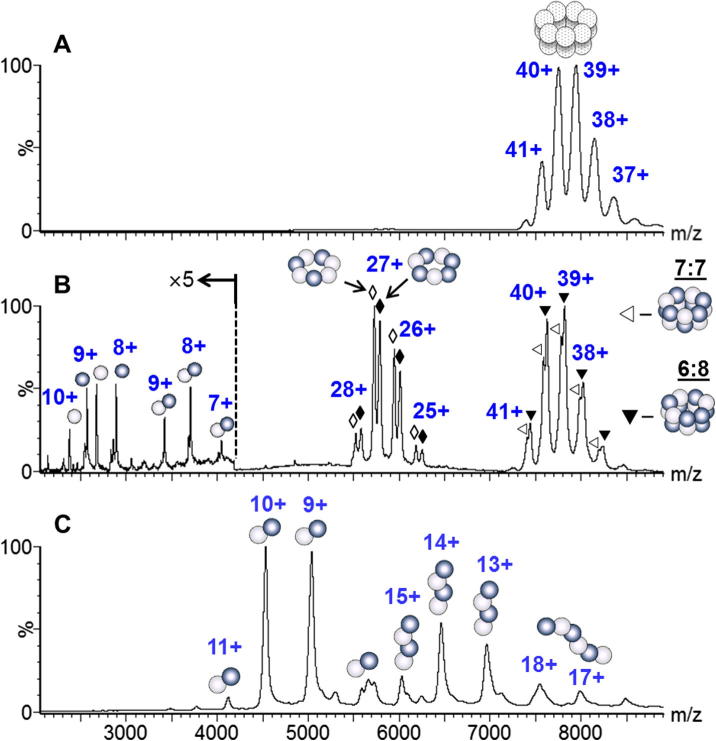
Nano-ESI mass spectra of ClpP1/ClpP2 protease. Charge states of the observed species are indicated by numbers. (A) Sample in 200 mM ammonium acetate, charge states of the tetradecameric double-ring complex observed with low resolution. (B) Same sample with 2 mM of TEA. Bright and dark balls represent ClpP1and ClpP2 subunits, respectively. Hollow diamonds (♢) and filled diamonds (♦) indicate charge states of 4ClpP1 + 3ClpP2 and 3ClpP1 + 4ClpP2 heptamers, respectively. Hollow triangles (▽) and filled triangles (▾) indicate charge states of (4ClpP1 + 3ClpP2)(3ClpP1 + 4ClpP2) and (3ClpP1 + 4ClpP2) × 2 tetradecameric double-ring complexes with ClpP1/ClpP2 relative stoichiometry of 7:7 and 6:8, respectively. Depicted arrangement of the subunits in the ring is one of a few possibilities (Fig. 6). (C) Same sample with 10 mM TEA and 2.5% of ammonium hydroxide: charge states of (ClpP1)(ClpP2) heterodimers, (ClpP1)_2_(ClpP2)_2_ heterotetramers and (ClpP1)_3_(ClpP2)_3_ heterohexamers are observed.

To determine the subunit arrangement of the ClpP1/P2 complex, we applied different mass spectrometry approaches. Peaks corresponding to the charge states of the double ring tetradecamer were clearly observed in mass spectra from the non-denaturing solution (Fig. 2A), highlighting the fact that, the electrospray conditions were even ‘softer’ than the native gel (Fig. 1C). The peaks in this spectrum are however broadened due to incomplete desolvation (Hernandez and Robinson, 2007), and the accuracy of the mass measurement (310 ± 20 kDa) is not sufficient to define the stoichiometry of the intact complex. To probe the arrangement of the subunits within the complex we used weak denaturing conditions (2 mM TEA). Under these conditions the mass spectra become more complicated exhibiting several groups of peaks assigned to charge states of monomers, heterodimers, heptamers and tetradecamers (Fig. 2B and Table 1). The mass resolution achieved under these solution conditions is higher than for the intact complex and allows us to assign the stoichiometry of both single and double ring complexes. Further denaturation leads to the dissociation of the rings producing hetero- dimers, tetramers and hexamers (Fig. 2C and Table 1).

### 3.2 Stoichiometry of the single ClpP1/P2 ring complex

A series of charges states assigned to the single ring complex is observed under weakly denaturing electrospray conditions (Fig. 2B, *m/z* = 5000–6500). Each charge state is split into doublets however with molecular masses of 157.20 and 158.93 kDa (Fig. 2B, hollow and filled diamonds). These double peaks are assigned to the ring stoichiometries of 4ClpP1 + 3ClpP2 and 3ClpP1 + 4ClpP2, respectively, in accord with the predicted molecular masses (Table 1). The intensities of these peaks are approximately equal, implying comparable population of complexes with both stoichiometries. Serial dilution of the protein complex demonstrated the same relative abundances of the two forms of the ring structure over a range of protein and TEA concentrations from 0.2 to 4 mg/ml, and from 1.0 to 12 mM respectively, confirming the approximately equal population of these two single ring complexes (data not shown).

**Fig. 3:**
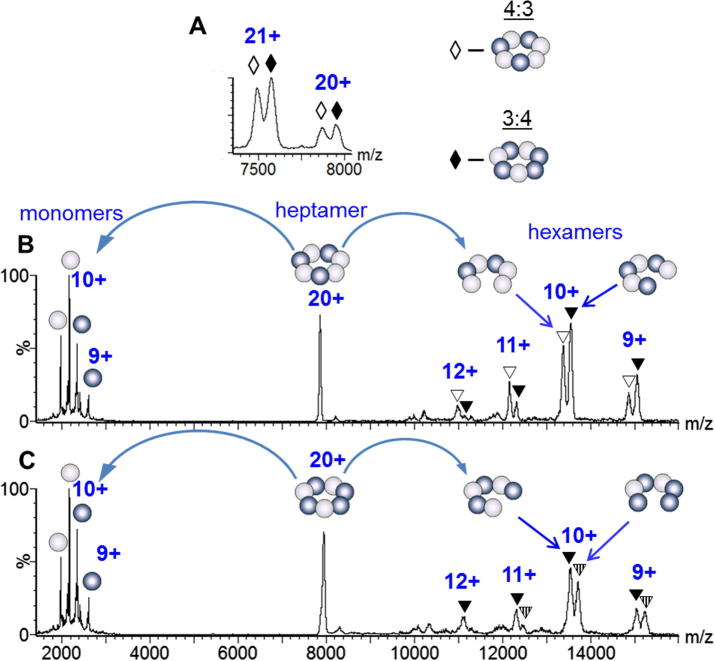
Mass spectra of ClpP1/ClpP2 heptamer sprayed from solution with 10 mM of TEA. Bright and dark balls represent ClpP1 and ClpP2 subunits, respectively. Depicted arrangement of the subunits is one of a few possibilities (Fig. 6). (A) Charge states of the heptamer ions: 4ClpP1 + 3ClpP2 and 3ClpP1 + 4ClpP2 are indicated with hollow (♢) and filled (♦) diamonds, respectively. (B) CID of the isolated 20+ charge state of 4ClpP1 + 3ClpP2 complex leads to formation of 4ClpP1 + 2ClpP2 (hollow triangles, ▽) and 3ClpP1 + 3ClpP2 (filled triangles, ▾) fragments through the loss of ClpP2 and ClpP1 subunits, respectively. (C) CID of the 20+ charge state of 3ClpP1 + 4ClpP2 complex leads to a similar loss of either subunit from the ring producing 3ClpP1 + 3ClpP2 (▾) and 2ClpP1 + 2ClpP2 (patterned triangles) fragments.

Further confirmation of the two single ring stoichiometries comes from collision induced dissociation (CID) mass spectra of isolated charge states (Fig. 3). When the 20+ charge state of the 4ClpP1 + 3ClpP2 complex was isolated and subjected to CID, the resulting fragment ions could be assigned to monomer subunits ClpP1 and ClpP2 recorded at *m/z* < 3000, and the corresponding hexameric fragments 4ClpP1 + 2ClpP2 and 3ClpP1 + 3ClpP2 recorded at *m/z* > 7000 (Fig. 3B and Table 2). Similarly, CID of the 20+ charge state of the ring with the other stoichiometry, 3ClpP1 + 4ClpP2 resulted in ClpP1 and ClpP2 monomeric subunits and the corresponding 3ClpP1 + 3ClpP2 and 2ClpP1 + 4ClpP2 hexameric complexes (Fig. 3C and Table 2). The experimental masses of the dissociation products from the single ring structures are in good agreement with the values predicted for the dissociation of 4ClpP1 + 2ClpP2 and 3ClpP1 + 4ClpP2 (Table 2), thus confirming the dual stoichiometry of the ring.

Interestingly, comparing the intensities of the two hexameric products reveals a greater population of the dissociation product 3ClpP1 + 3ClpP2 forming from 3ClpP1 + 4ClpP2 in line with the higher probability of dissociating a ClpP2 subunit when four are present (Fig. 3C). The analogous situation occurs when ClpP1 is the predominant subunit (Fig. 3B) confirming the equal probability of the two subunits to dissociate from single rings.

### 3.3 Stoichiometry of the double ring complex

To form the double ring complex there are three possible combinations: two copies of the lower mass ring (LMR) 4ClpP1 + 3ClpP3: (LMR + LMR); one copy of LMR and one of the higher mass ring (HMR) 3ClpP1 + 4ClpP2: (LMR + HMR); and two copies of HMR (HMR + HMR), Table 3. Assuming equal populations of both types of single ring and equal propensity to form the double ring, either with itself or the other type of ring, all three stoichiometries should be present with relative abundances of 1:2:1 (Table 3). Mass spectra of the double ring species reveal charge states of these tetradecameric complexes with at least two stoichiometries (Fig. 2B). The two complexes can be assigned as LMR + HMR and HMR + HMR (Table 3). Surprisingly the other possible combination of the two rings, LMR + LMR with a theoretical mass of 314.24 kDa is not apparent in the mass spectrum. Given the broad nature of the peaks observed for the double-ring complexes, however it is not possible to rule out entirely the presence of a low population of LMR + LMR.

**Fig. 4:**
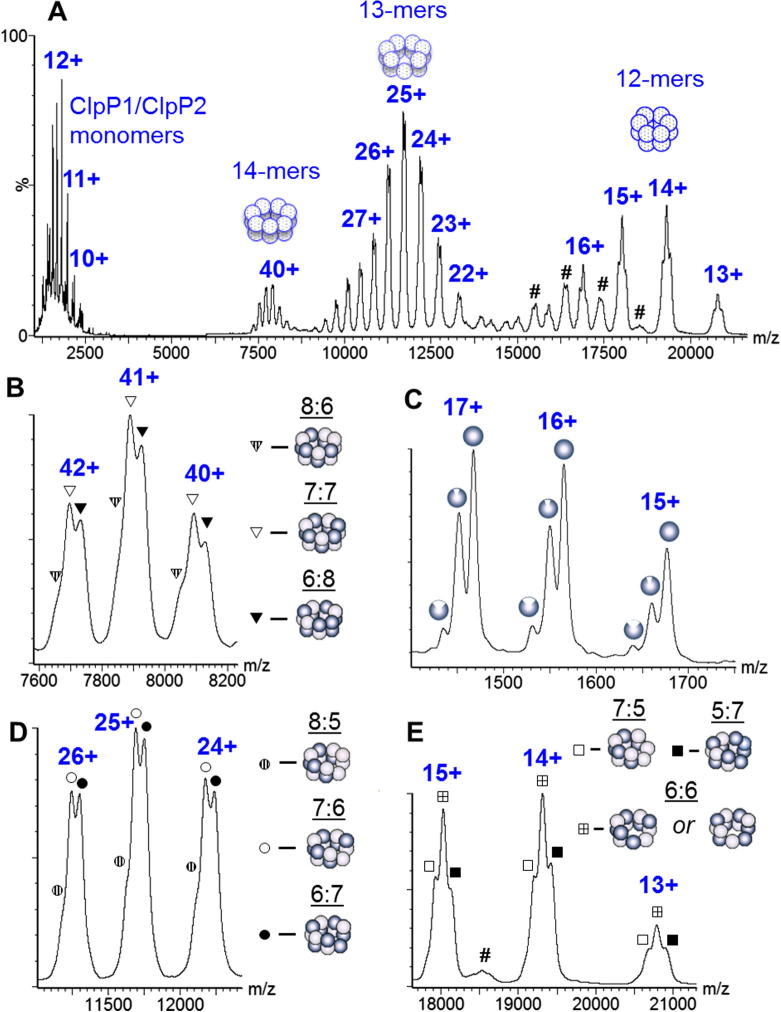
CID mass spectra of all charge states of ClpP1/ClpP2 tetradecamer from ammonium acetate solution without TEA. (A) Full range mass spectrum taken at a collision voltage of 175 V. Hashes (#) label fragments produced by partial protein backbone cleavage. (B) Expanded view over the charge state peaks of tetradecameric double rings: patterned, hollow and filled triangles indicate LMR·LMR, LMR·HMR and HMR·HMR complexes with ClpP1/ClpP2 stoichiometry of 8:6, 7:7 and 6:8, respectively. Bright and dark balls represent ClpP1 and ClpP2 subunits, respectively. (C) Low-mass fragment ions produced at a collision voltage of 50 V are mostly ClpP2 monomers (whole dark balls) and their fragments (dented balls). (D) Expanded view over the charge state peaks of tridecamer fragment ions. Hollow (○) and filled circles (●) indicate charge states of tridecamers produced via loss of ClpP2 from LMR·HMR and HMR·HMR complexes with 7:6 and 6:7 stoichiometry, respectively. Patterned circles indicate features assigned to tridecamers produced via the loss of ClpP2 from LMR·LMR complex with 8:5 stoichiometry. (E) Expanded view over peaks of dodecameric ion fragments. Patterned, hollow and filled squares indicate charge states of the 269.1, 270.84 and 272.58 kDa dodecamers with assigned stoichiometry of 7:5, 6:6 and 5:7, respectively (Table 2). Depicted arrangement of the subunits in the ring is one of a few possibilities (Fig. 6).

To probe further the stoichiometries of the double ring tetradecameric complexes we carried out CID of all charge states observed from non-denaturing solution conditions and initially without mass selection (Fig. 4). This procedure led to an increase in resolution together with an unresolved peak at the low *m/z* side of each charge state, indicating the presence of a third stoichiometry with a lower molecular mass (Fig. 4B). This species was tentatively assigned to the LMR + LMR assembly (Table 3).

Additionally, we carried out CID of the double ring tetradecamer which led to losses of single subunits from the complex producing monomeric and tridecameric fragments and, at higher collision energies, dodecameric fragments (two subunits lost from the complex in sequence), Fig. 4A. To assign these product ions the stoichiometries of the tridecameric and dodecameric fragments were derived from the precursor tetradecamers based upon the assumption that loss of either ClpP1 or ClpP2 could occur from the complex with equal probability upon CID (Table 3). However, at low collision energy, when only tridecameric and no dodecameric products were formed, the only monomeric species observed were ClpP2 ions (Fig. 4C). Their partner tridecameric fragments must therefore be produced by loss of ClpP2 rather than ClpP1 from the double-ring complex. In accord with this, no tridecameric fragments corresponding to the loss of ClpP1 from HMR + HMR ring were observed (Fig. 4D and Table 3). Furthermore, comparison of the peaks corresponding to the charge states of tridecamers (Fig. 4D) with those of tetradecamers (Fig. 4B) reveals a similarity of their peak splitting that would be expected if the 13-mers were produced from the 14-mers *via* mass and charge reduction associated with a loss of a single ClpP2 from each of the three possible stoichiometries of the double ring. Molecular masses of the 13-mers are in accord with the masses predicted for the double-ring products after extraction of one ClpP2 (Table 3). Therefore we conclude that the 14-mer has two predominant stoichiometries: LMR + HMR and HMR + HMR, and one much less abundant: LMR + LMR.

12-mers are produced by further dissociation of 13-mers at high collision voltages (Fig. 4A and E). At least three 12-mers can be identified and assigned to loss of either ClpP1 or ClpP2 from the two most prominent 13-mers (Fig. 4E and Table 3). Theoretical masses for these fragments are in accord with experimental values, and the 270.84 kDa 12-mer is predicted to be the most abundant since two 13-mers (292.54 and 294.28 kDa) can contribute to its production (Table 3, Fig. 4E crossed squares). Additionally, complexes in which the protein backbone is cleaved are also observed at high collision energy (MW *ca.* 278 kDa) (Fig. 4A hashes). Similar results have been obtained from the mass selected CID of particular charge states of the double-ring complex (data not shown).

### 3.4 Subunit connectivity in the ring

As the Clp rings have 3:4 or 4:3 stoichiometry with respect to ClpP1 and ClpP2 subunits, at least two of the four identical subunits in the ring must be connected. Inferring the precise arrangement of the other subunits in the ring requires the mass analysis of sub-oligomers produced by partial dissociation of the heptameric rings, either in solution or in the gas phase. As shown above, gas-phase CID of the ClpP1/P2 heptameric ring only leads to the loss of a single subunit and resulted in the monomeric and hexameric ion fragments *via* asymmetric dissociation (Fig. 3). The (ClpP1)(ClpP2) heterodimer was frequently observed in mass spectra of solutions containing denaturing agent (Fig. 2B and C), but neither (ClpP1)_2_ nor (ClpP2)_2_ homodimer was observed. At some denaturing conditions (10 mM TEA and 2.5% ammonium hydroxide) when the ring heptamers were almost completely dissociated, more sub-oligomers peaks were observed and assigned to heterodimers, heterotetramers (ClpP1)_2_(ClpP2)_2_ and heterohexamers, (ClpP1)_3_(ClpP2)_3_, Fig. 2C. Importantly, complete dissociation of the heptamer did not produce (ClpP1)_4_/(ClpP2)_4_ homotetramers, or (ClpP1)_3_(ClpP2)/(ClpP1)(ClpP2)_3_ heterotetramers_,_ which would only result from a ring where three or four subunits of the same type, ClpP1 or ClpP2, were adjacent in the ring. Such arrangements of subunits in the ring are therefore excluded.

**Fig. 5:**
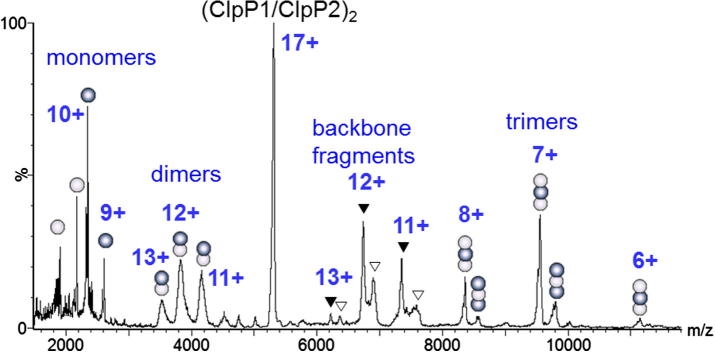
CID MS/MS spectra of the 17+ charge state of the (ClpP1/ClpP2)_2_ heterotetramer from the solution with 10 mM TEA and 1.5% of ammonium hydroxide. Bright and dark balls represent ClpP1 and ClpP2 subunits, respectively. Filled (▾) and hollow (▽) triangles label charge states of the complexes containing fragmented subunits (MW 80.8 and 82.7 kDa, respectively).

The dissociation products can be summarized as (ClpP1/ClpP2)*_n_*, where *n* = 1, 2 and 3. No dissociation products containing unequal numbers of ClpP1 and ClpP2 subunits (*i.e.* heterotrimers or heteropentamers) were recorded from the denaturing solution. Therefore, under denaturing conditions the additional lone subunit dissociates from the complex more readily than the heterodimers. Further dissociation of the ring proceeds through breaking the non-covalent interactions between the dimers thus prompting the formation of (ClpP1/ClpP2)_2_ and ClpP1/ClpP2. Collision induced dissociation of (ClpP1/ClpP2)_2_ heterotetramers results in ClpP1, ClpP2 monomers, ClpP1/ClpP2 heterodimers, (ClpP1)_2_(ClpP2) and (ClpP1)(ClpP2)_2_ heterotrimers, and complexes containing backbone fragments of either subunit (Fig. 5). The absence of homodimers and presence of heterodimers among the products from CID of (ClpP1/ClpP2)_2_ tetramer also indicate the importance of ClpP1/ClpP2 heterodimers in the organization of the ring.

**Fig. 6:**
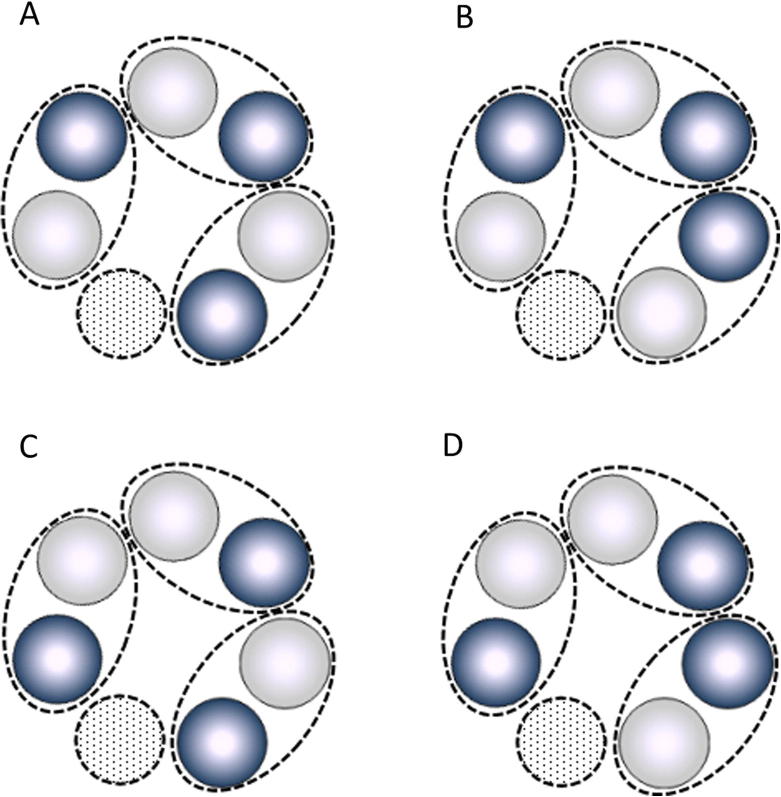
Possible arrangements of ClpP1 and ClpP2 subunits in the heptameric ring. Bright and dark balls represent ClpP1 and ClpP2 subunits, respectively, dotted circles represent either ClpP1 or ClpP2 ‘sealing’ subunit. Dotted elliptic lines around pairs of subunits indicate the borders of ClpP1/ClpP2 heterodimers.

Together these results indicate that ClpP1/ClpP2 heterodimers play an important role in the formation of the heptameric ring of the protease. The ring appears therefore to be formed from the association of three dimers, with the resulting (ClpP1/ClpP2)_3_ hexamer interacting with a lone ClpP1 or ClpP2 subunit to form a heptameric ring. Four arrangements of the ClpP1/ClpP2 dimers in the heptameric ring are possible (Fig. 6).

In one of the possible ring structures, ClpP1 and ClpP2 subunits are arranged in an alternating order with the exception of the seventh subunit that ‘completes’ the ring (Fig. 6A). This is analogous to the order of subunits in the ClpP3/ClpR complex that we described previously (Andersson et al., 2009). In the other three possible arrangements there are one or two pairs of adjacent identical subunits in the (ClpP1/ClpP2)_3_ hexamer within the ring.

## 4 Discussion and conclusion

ClpXP1/P2 is one of two different Clp proteases that function in *Synechococcus* under standard growth conditions. In this study we have purified the *Synechococcus* ClpP1/P2 proteolytic core by dual over-expression in *E. coli*. The two recombinant ClpP proteins have the same size as their native counterparts, which in the case of ClpP2 also confirmed it correctly processed autolytically its N-terminal extension. As used previously when purifying the *Synechococcus* ClpP3/R complex (Andersson et al., 2009), the His_6_ affinity tag was included only on one of the subunit types, which demonstrated that ClpP1 and ClpP2 readily assembled together into a mixed oligomer. The size of the mixed oligomer as resolved by CN-PAGE matched that of the native ClpP1/P2 heptamers (Stanne et al., 2007). The recombinant ClpP1/P2 core also corresponded to the native complex functionally by being proteolytically active with ClpX but not ClpC, consistent with its known chaperone specificity *in vivo* (Stanne et al., 2007, Ståhlberg et al., 2015) To further investigate the subunit arrangement of the ClpP1/P2 proteolytic core we used non-denaturing mass spectrometry. This revealed that the ClpP1/P2 complex had equal ratios of the two different paralogs, which matched the equimolar concentrations of ClpP1 and ClpP2 in *Synechococcus* under standard growth conditions (Ståhlberg et al., 2015). The recombinant ClpP proteins formed two different heptameric rings with either a 3:4 or 4:3 ratio of ClpP1:ClpP2 subunits. The different heptameric rings assemble primarily into two distinct tetradecameric proteolytic cores, with either (4ClpP1 + 3ClpP2) + (3ClpP1 + 4ClpP2) or 2 × (3ClpP1 + 4ClpP2), with most of the subunits in both configurations alternating from one paralog to the next. The other possible combination of the rings, 2 × (4ClpP1 + 3ClpP2) is less populated than the other two. The only other example of such a heterologous Clp proteolytic core is the *Synechococcus* ClpP3/R. Like ClpP1/P2, the ClpP3/R core consists of heptameric rings with mixed subunits in a 4:3 ratio and arranged in an alternating configuration (Andersson et al., 2009). However, the organization of the ClpP3/R core is less variable, with only one type of heptameric ring observed with a 4ClpR + 3ClpP3 stoichiometry. Furthermore, as evident from our data on partially denatured ClpP1/P2 complexes, ClpP1/ClpP2 heterodimers play a more important role in organizing the structure, than in the case of ClpP3/R core. It remains unclear why the two constitutive Clp proteases in *Synechococcus* consists of such proteolytic cores with mixed subunit types, although given their conservation this appears to be a common features of these enzymes in cyanobacteria.

Although the type of mixed Clp proteolytic cores typified by ClpP1/P2 and ClpP3/R is so far unique to cyanobacteria and other photosynthetic organisms, another variation can be found in certain non-photosynthetic bacteria. In this type, the core complex consists of two different ClpP paralogs also termed ClpP1 and ClpP2 but in this case they assemble into separate homogeneous heptamers. In *Listeria monocytogenes*, the LmClpP2 protein can form its own tetradecamer that is proteolytically active, whereas LmClpP1 only forms inactive heptameric rings by itself. However, when both proteins are co-expressed together they form a proteolytic core with one heptameric ring of LmClpP1 and one ring of LmClpP2. Within this complex, both LmClpP1 and LmClpP2 are proteolytically active, although the activity of LmClpP1 is dependent on its association to the LmClpP2 ring (Zeiler et al., 2011, Zeiler et al., 2013). In comparison, the two ClpP orthologs in *M. tuberculosis*, MtClpP1 and MtClpP2 can individually form their own tetradecamer *in vitro* but both complexes are proteolytically inactive (Personne et al., 2013). It is only when both the recombinant MtClpP1 and MtClpP2 proteins are combined that they oligomerize into a proteolytically active complex, although this requires the presence of an activator peptide (N-blocked peptide aldehydes). Like the LmClpP1/P2 core, the one formed by MtClpP1 and MtClpP2 consists of one heptameric ring of MtClpP1 and one of MtClpP2 (Akopian et al., 2012).

In a parallel study, we have recently shown that both ClpP1 and ClpP2 contribute to the catalytic activity of the ClpP1/P2 proteolytic core. However, the alternating arrangement of ClpP1 and ClpP2 subunits appears to limit the catalytic activity of the core complex, with inactivation of either ClpP1 or ClpP2 increasing rather than decreasing the overall proteolytic rate of the tetradecamer (Ståhlberg et al., 2015). A similar observation has recently been made for the MtClpP1/P2 complex (Schmitz and Sauer, 2014) suggesting that the two types of mixed Clp proteolytic cores might be similarly regulated. The catalytic rate of the essential ClpP3/R protease in *Synechococcus* might also be controlled in a similar manner. We have shown that if the catalytic site is restored to the normally inactive ClpR subunit that the proteolytic activity of the resulting ClpP3/R core is no faster than the original one (Andersson et al., 2009, Tryggvesson et al., 2012), demonstrating that the presence of neighboring active subunits somehow restricts proteolytic activity. Although the advantage underlying the modification of the Clp proteolytic cores to include mixed subunits remains unclear, this phenomenon has been observed for Clp proteases in many different bacteria including cyanobacteria as well as to more extreme diversity in the enzymes in the chloroplasts of algae and plants that can contain up to eleven distinct subunit types. Why such diversity exists, will probably only begin to be understood following high resolution structure determination of these mixed Clp complexes, their fine structural details likely revealing the evolutionary advantage of this mixed proteolytic core arrangement.

## Figures and Tables

**Table 1:** Assignment of the mass peaks observed directly from electrosprayed sample solutions.

Experimental mass/kDa	Assignment	Theoretical mass/kDa
21.76	ClpP1	21.70
23.53	ClpP2	23.44
45.32	ClpP1 + ClpP2	45.14
157.20	4ClpP1 + 3ClpP2	157.12
158.93	3ClpP1 + 4ClpP2	158.86
316.28	(4ClpP1 + 3ClpP2) × (3ClpP1 + 4ClpP2)	315.98
317.79	2×(4ClpP1 + 3ClpP2)	317.72
90.47	2ClpP1 + 2ClpP2	90.28
135.53	3ClpP1 + 3ClpP2	135.42

**Table 2:** Stoichiometries of Clp protease heptamers and possible hexamer fragments from them. Experimental masses from CID MS of the heptamer 20+ charge state are given in round brackets.

Ring stoichiometry	MW/kDa	Monomer fragment	Hexamer fragment	Molecular weight/kDa
4ClpP1 + 3ClpP2	157.12	ClpP2 (23.46 kDa)	4ClpP1 + 2ClpP2	133.68 (133.84)
4ClpP1 + 3ClpP2	157.12	ClpP1 (21.72 kDa)	3ClpP1 + 3ClpP2	135.42 (135.52)
3ClpP1 + 4ClpP2	158.86	ClpP2 (23.50 kDa)	3ClpP1 + 3ClpP2	135.42 (135.51)
3ClpP1 + 4ClpP2	158.86	ClpP1 (21.77 kDa)	2ClpP1 + 4ClpP2	137.16 (137.17)

**Table 3:** Expected double-ring stoichiometries and sub-oligomeric fragments from them. LMR stands for the low mass ring, 4ClpP1 + 3ClpP2, and HMR stands for the high mass ring, 3ClpP1 + 4ClpP2. Relative ClpP1/P2 stoichiometries are given in square brackets. Experimental masses are given in round brackets. Question mark (?) indicates the fragments not recorded in the mass spectra.
